# Productivity cost due to postpartum ill health: A cross-sectional study in Sri Lanka

**DOI:** 10.1371/journal.pone.0185883

**Published:** 2017-10-11

**Authors:** Nuwan Darshana Wickramasinghe, Jennifer Horton, Ishani Darshika, Kaushila Dinithi Galgamuwa, Wasantha Pradeep Ranasinghe, Thilini Chanchala Agampodi, Suneth Buddhika Agampodi

**Affiliations:** Department of Community Medicine, Faculty of Medicine and Allied Sciences, Rajarata University of Sri Lanka, Anuradhapura, Sri Lanka; University of Bristol, UNITED KINGDOM

## Abstract

**Objective:**

Even though postpartum morbidity continues to cause high disease burden in maternal morbidity and mortality across the globe, the literature pertaining to resultant productivity loss is scarce. Hence, the present study aimed at determining the productivity loss and associated cost of episodes of postpartum ill health.

**Methods:**

A cross sectional study was conducted in two Medical Officer of Heath areas in the Anuradhapura district, Sri Lanka in 2011, among 407 women residing in Anuradhapura district with an infant aged between 8 to 24 weeks. Validated interviewer administered questionnaires, including the IMMPACT productivity cost tool, were used to collect data on self-reported episodes of postpartum ill health. The productivity loss was calculated as the sum of days lost due to partial and total incapacitation. The adjusted productivity loss for coping strategies was calculated. Productivity cost, both total and adjusted, were calculated based on the mean daily per capita income of the study sample.

**Results:**

Of the 407 participants, 161(39.6%) reported at least one episode of postpartum illness. Hospitalisations were reported by 27 (16.8%) of all symptomatic postpartum women. Common symptoms of postpartum ill health were pain/infection at either episiotomy or surgical site (n = 44, 27.3%), lower abdominal pain (n = 40, 24.8%) and backache (n = 27, 16.8%). The mean productivity loss per episode of ill health was 15 days (SD = 7.8 days) and the mean productivity loss per episode after adjusting for coping strategies was 7.9 days (SD = 4.4 days). The mean productivity cost per an episode was US$ 34.2(95%CI US$ 26.7–41.6) and the mean productivity cost per an episode after adjusting for coping strategies was US$ 18.0 (95%CI US$ 14.1–22.0)

**Conclusions:**

The prevalence of self-reported postpartum ill health, associated productivity loss and cost were high in the study sample and the main contributors were preventable conditions including pain and infection. Thus, effective pain management and proper infection prevention and control measures are important in reducing the burden of postpartum illness and resultant productivity cost.

## Introduction

Since maternal and child health is recognised as one of the major areas in the global health agenda with consequent concerted multi-stakeholder efforts to improve maternal health across the globe, the total annual estimated number of global maternal deaths declined from 376,034 in 1990 to 292,982 in 2013 with a substantial declining rate of 3.3% in 2012–13 period [1,2]. Notwithstanding the significant achievements in relation to the reduction in maternal mortality, it is estimated that for each maternal death, approximately 6.2 women experience severe complications from pregnancy and childbirth [2]. Furthermore, 7.3% of all pregnant women experience potential life-threatening events during pregnancy and childbirth [2]. In terms of disease burden, maternal morbidities are cited as the leading cause of Disability-Adjusted Life Years (DALYs) lost among women in reproductive age group in the developing countries [3]. Furthermore, WHO has estimated that 50 million incidents of pregnancy-related complications have resulted in an annual loss of nearly 40 million DALYs [4].

In comparison to the plethora of research pertaining to maternal morbidities during pregnancy, there is a relative paucity of literature pertaining to postpartum morbidities. Most of the small body of research on postpartum outcomes in the developing world is focused on the “near-misses”, immediately surrounding birth including postpartum haemorrhage, retained products, fistula and uterine prolapse [5, 6]. Due to the predominance of institution based studies, relatively minor ailments during the postpartum period which do not necessitate hospitalisation, tend to be far less documented. Though, the postpartum conditions present within the first 42 days after delivery, their impact on daily function and productivity may extend much longer [7]. Greater focus paid on maternal mortality reduction and averting maternal near-misses has served its purpose during the last few decades, which is evident by the improvement in maternal mortality indicators. Despite these improvements, the goal of improving maternal health is identified as a main challenge in global health agenda [8].

Sri Lanka, despite being a lower middle income country, has exceptional achievements with regard to maternal and child health indicators, thus, Sri Lankan case study is often cited in global maternal health agenda as a cost effective and highly successful programme which reduced maternal mortality by 50% within 10–12 years [9]. Well established maternal care service delivery system up to the grass root level, properly structured maternal mortality surveillance system and a combined system of community based verbal autopsy and institutional based confidential inquiry in Sri Lanka are regarded as crucial factors in bringing down the maternal mortality. The maternal mortality ratio in Sri Lanka is 32.5 per 100,000 live births and out of the total maternal deaths, 60% of deaths have occurred during the postpartum period [10]. Despite, the existence of a well structured maternal mortality surveillance system, the surveillance of postpartum health conditions in Sri Lanka is not yet well developed. According to the 2013 statistics, the reported new cases of postpartum morbidities in Sri Lanka amounts to 11.5% of total reported deliveries [10]. On the contrary, a study conducted in Kalutara district in Sri Lanka, has revealed that only 11% of postpartum women in the study sample did not have any signs or symptoms of ill health [11]. While women receive postpartum care at domiciliary level by the Public Health Midwives (PHMM), anecdotally, the visits tend to focus more on breastfeeding and infant care issues. This limits time and resources available to address other concerns of maternal wellbeing [12, 13, 14].

Amongst the available literature related to postpartum morbidities, the studies conducted with regard to quantification of productivity loss and associated productivity cost is scanty [3]. Furthermore, there is a paucity of evidence about mild to moderate postpartum morbidities experienced by women in the developing world and little understanding of how this impacts daily function and productivity. Thus far, there is no published literature available in that regard in the Sri Lankan context. The studies conducted in relation to antenatal maternal morbidities, under the research project titled “Disease Burden and the Economic Impact of Maternal Morbidity in Sri Lanka”, revealed that the leading causes of productivity loss are not major illnesses or maternal near misses, but minor ailments in pregnancy [15, 16].According to the study findings, minor ailments such as nausea and vomiting during pregnancy and backache were the commonest ill health conditions experienced by antenatal mothers in Anuradhapura district, Sri Lanka [16] and the mean productivity cost due to the last episode of ill health among antenatal mothers were reported as LKR 8,444.26 [15]. However, literature pertaining to productivity cost among postpartum mothers is not available in Sri Lanka.

Given the vacuum of research pertaining to disease burden and economic burden of postpartum morbidities, the present study was designed with the objective of determining the productivity loss and productivity cost due to postpartum morbidities in a selected area of Sri Lanka.

## Materials and methods

### Study design and setting

A descriptive cross sectional study was conducted in the Anuradhapura district, located in the North Central Province of Sri Lanka, from January 2011 to April 2011. This study was conducted as one arm of the Maternal Morbidity Project in the Anuradhapura district, titled “Disease Burden and the Economic Impact of Maternal Morbidity in Sri Lanka” [15, 16]. Ethical clearance for the study was obtained from the Ethics Review Committee of Faculty of Medicine and Allied Sciences, Rajarata University of Sri Lanka.

The total population residing in the Anuradhapura district in 2010 was 886,945, out of which 92.7% were living in the rural sector. Anuradhapura district has19 public health divisions known as Medical Officer of Health (MOH) areas and each MOH area is divided into sub-divisions known as Public Health Midwife (PHM) areas with a population ranging from 1,500 to 3,000. In each PHM area the maternal and child health service are provided through the area PHM.

### Participants

The study population consisted of women residing in Anuradhapura district with an infant aged more than 8 weeks to less than 24 weeks. Since the study aimed at detecting the self-reported episodes of postpartum ill health, women with infants aged more than 24 weeks were not included to minimise recall bias. The required sample size for the study was calculated so as to be able to make estimates with a 95% confidence level and 0.05 precision. Owing to the wide variation of reported estimates of the prevalence of postpartum morbidities in Sri Lanka [10, 11], the anticipated prevalence of postpartum ill health was taken as 50% to maximise the sample size, which yielded a sample size of 384.

Two MOH areas in the Anuradhapura district were randomly selected and in the two MOH areas, eligible postpartum women in all PHM areas were invited to participate in the study via the area PHMM. Subject recruitment was done upon the informed written consent of the participants at field child immunisation clinics. Since almost all postpartum women accompany their infants to field immunisation clinics and the infant immunisation coverage is 99% in the Anuradhapura district, this sampling procedure was adopted for the study [17].

### Data collection

Data collection was carried out by five Medical graduates. Prior to data collection, all data collectors were given a comprehensive training on data collection procedures, use of protocols, probing and extracting data from records. During the pilot study and pre-testing, issues related to data collection, data quality and data incompatibility were discussed. A validated interviewer administered questionnaire was used as the study instrument, which included the IMMPACT productivity cost tool. Translation and cultural adaptation of this tool was done using qualitative and quantitative methods as described previously [15].

The interviewer administered questionnaire consisted of questions pertaining to basic socio demographic data of study participants. In order to assess the episodes of self-reported postpartum ill health, a comprehensive list of the related symptoms identified through a thorough literature review and a series of focus group discussions with postpartum mothers and PHMM, was included in the questionnaire.

Data on the symptoms of the last ill health episode, duration of illness, frequency of illness, treatment sought, hospitalisation and its associated frequency and duration were collected. For the calculation of productivity loss, the effect of the episodes of ill health on daily activities and data on coping strategies were gathered. Coping strategies to compensate productivity loss were categorised as intra-household adaptation (the help of a household member to carry out daily activities), adaptation involving social networks (the help of a relative/friend/neighbour to carry out daily activities) and hired assistance during the episodes of ill health. In the validated IMMPACT productivity cost tool, the effect on daily activities was measured using a visual analog scale of 0 to 5; ‘0’ representing total incapacitation due to illness and ‘5’ representing no effect. Verification of extracted data on episodes of reported ill health was done by using available documented evidence, such as diagnosis cards and patient records, whenever appropriate.

### Data analysis

The episodes of different postpartum ill health were calculated as proportions out of the total study sample. Productivity loss attributed to the episodes of ill health was calculated as partial incapacitation and total incapacitation as per the guidelines of the IMMPACT tool kit [18]. Coping strategies were identified based on the information gathered in relation to the assistance received by the postpartum mothers for daily activities during the episodes of ill health. The amount of assistance received for daily activities during the episodes of ill health were quantified as recovered/compensated productivity loss (in days). The adjusted productivity loss for coping strategies was also computed by using the estimates of total productivity loss and recovered productivity loss.

Translation of the productivity loss into monetary terms for the purpose of calculating the productivity cost was done based on the mean daily per capita income. The per capita income was calculated on the basis of equal weightage given to all household members and under the assumption that work carried out by postpartum women is equally important in contributing to the household income, as previously discussed [15]. The equivalent monetary values for the estimates in US Dollars (US$) were computed based on the conversion rate of LKR 147.32 per US$ 1.

## Results

### Sample characteristics

The sample consisted of 407 postpartum women in two MOH areas in the Anuradhapura district. Sample consisted primarily of Sinhalese women (n = 396, 97.3%). Mean age of the study sample was 27.7years (SD 5.3 years) (Table 1). The majority of women were 8–12 weeks postpartum (n = 196, 48.2%) at the time of the study and 77.6% (n = 316) of participants were educated up to General Certificate of Examination Ordinary Level (Grade 11).

**Table 1:** Characteristics of the study sample (n = 407).

Characteristic	Number	Percentage
**Age**		
<20 years	20	4.9%
20–27 years	174	42.8%
28–35 years	180	44.2%
**Education level**		
Up to grade 5	14	3.4%
Up to grade 10	188	46.2%
Up to G.C.E. Ordinary Level	114	28.0%
Completed G.C.E. Advanced Level	61	15.0%
Tertiary education	30	7.4%
**Monthly family income**		
Less than LKR 20,000	80	19.7%
LKR 20,001–40,000	193	43.4%
LKR 40,001–60,000	72	17.7%
LKR 60,001–80,000	24	5.9%
LKR 80,001–100,000	18	4.4%
More than LKR 100,001	20	4.9%
**Weeks postpartum**		
8–12 weeks	196	48.2%
13–18 weeks	97	23.8%
18–24 weeks	114	28.0%
**Mode of delivery**		
Vaginal (Normal vaginal/assisted)	309	75.9%
Lower Segment Caesarean Section	98	24.1%
Total	407	100.0%

G.C.E., General Certificate of Examination; LKR, Sri Lankan Rupees

Most women were engaged primarily in a care giving role, with 83.0% (n = 338) listing their primary occupation before the childbirth as housewife. Those employed prior to child birth, consisted mainly of professionals or technical trades (4.4%), clerical workers (4.2%) and skilled labourers (4.9%). The majority of women (n = 273, 63.1%) were from families with a monthly family income of less than LKR 40,000.00. The mean monthly family income of the study sample was LKR 42,753.97 (SD LKR 49,250.82), which is equivalent to US$ 290.2 (SD US$ 334.31). The median number of household members in the study sample was 4 (IQR 4–5). The computed mean daily per capita income for the study sample was LKR 335.20 (SEM LKR 133.83),which is equivalent to US$ 2.3 (SEM US$ 0.9).

### Episodes of postpartum ill health

Of the 407 participants, 161 (39.6%) reported episodes of ill health during their postpartum period (Table 2). Commonest symptom of postpartum ill health was lower abdominal pain (n = 40, 24.8%), which was followed by backache (n = 27, 16.8%) and pain or infection of episiotomy site (n = 25, 15.5%).

**Table 2:** Episodes of postpartum ill health among postpartum women in Anuradhapura, Sri Lanka.

Symptom	Number experienced		Sought medical care		Admitted to hospital		Number of days in hospital	
	n	%	n	%^1^	n	%^1^	n	%^2^
Pain/Infection at LSCS site	19	11.8	8	5.0	5	3.1	15	10.9
Backache	27	16.8	14	8.7	5	3.1	22	16.1
Lower abdominal pain	40	24.8	19	11.8	3	1.9	15	10.9
Pain/Infection at episiotomy site	25	15.5	15	9.3	3	1.9	14	10.2
Vaginal bleeding	9	5.6	4	2.5	2	1.2	6	4.4
Fever	11	6.8	9	5.6	3	1.9	23	16.8
Breast engorgement	8	5.1	3	1.9	-	-	-	-
Dyspnoea	3	1.9	1	0.6	2	1.2	4	2.9
Headache	2	1.2	-	-	-	-	-	-
Hypertension	4	2.5	4	2.5	1	0.6	18	13.2
Leg swelling	2	1.2	-	-	-	-	-	-
Mastitis	2	1.2	2	1.2	2	1.2	10	7.3
Heartburn	1	0.6	1	0.6	-	-	-	-
Upper abdominal pain	1	0.6	-	-	-	-	-	-
Nausea/Vomiting	2	1.2	2	1.2	-	-	-	-
Dizziness	1	0.6	1	0.6	-	-	-	-
Other	4	2.5	1	0.6	1	0.6	10	7.3
Total	161	100.0	84	52.2	27	16.8	137	100.0

^1^ Percentage was calculated out of women with reported episodes of ill health (n = 161)

^2^ Percentage was calculated out of total number of hospital days (n = 137)

LSCS, Lower Segment Caesarean Section

Of the 161 postpartum women who reported episodes of acute ill health, 84 (52.2%) women sought medical treatment (Table 2). Hospitalisations were reported only by 27 (16.8%) of all symptomatic postpartum women. A total of 137 days were spent in the hospital by all women for postpartum ill health, with a mean of 5.1 days per admission. The leading cause of hospitalisation was pain or infection at Lower Segment Caesarean Section (LSCS) site, accounting for 18.5% (n = 5) of admissions and 10.9% (15 days) of total days spent in hospitals. However, the leading cause of lengthy hospital stays was fever (16.8% of total days in hospital), as measured by total admission days.

### Productivity loss due to postpartum ill health

Out of those who reported episodes of ill health, the majority (n = 110, 68.3%) reported the illness had negatively impacted their daily activities. Severe limitation of daily activities, i.e. total incapacitation was experienced by 50 (31.1%).

Partial incapacitation was mostly reported by women with symptoms of pain or infection at LSCS site (203.8 days, 21.8%). The other main contributors for work limitation were lower abdominal pain (179 days, 19.2%), backache (138.2 days, 14.8%) and pain or infection at episiotomy site (95.2 days, 10.2%).

The main symptoms of postpartum ill health resulted in total incapacitation were backache (384 days, 25.9%), pain or infection at LSCS site (333days, 22.5%) and pain or infection at episiotomy site (210 days, 14.2%).

The computed mean productivity loss per episode of postpartum ill health in the sample was 15 days (SD = 7.8days). In addition, the mean productivity loss per episode of pain/infection at LSCS site was 28.3 days and the corresponding figure for an episode of backache was 19.3 days.

The majority of women with an episode of ill health (n = 117, 72.7%) had received assistance with their daily activities (Table 3). The most common source of assistance was intra-household adaptation, i.e., the help of a household member to carry out daily activities, for 104 women (64.6%). Adaptation involving social networks in the form of the help of a relative/friend/neighbour to carry out daily activities was reported by twelve women (7.4%). Only a single woman received hired assistance (0.7%). The received assistance allowed for the net gain of 1141.6 day equivalents (47.2% of the productivity loss).

**Table 3:** Productivity loss due to episodes of ill health among postpartum women in Anuradhapura, Sri Lanka.

Symptom	Episodes	Total productivity loss (days)			Mean Productivity loss(days)	Number received assistance	Days compensated by coping strategies	Adjusted productivity loss (days)	Mean Adjusted productivity loss (days)
		Partial Incapacitation	Total incapacitation	Total					
Pain/Infection at LSCS site	19	203.8	333.0	536.8	28.3	19	178.8	358.0	18.8
Backache	27	138.2	384.0	522.2	19.3	16	217.0	305.2	11.3
Lower abdominal pain	40	179.0	197.0	376.0	9.4	24	164.0	212.0	5.3
Pain/Infection at episiotomy site	25	95.2	210.0	305.2	12.2	18	210.0	95.2	3.8
Vaginal bleeding	9	52.6	147.0	199.6	22.2	7	124.2	75.4	8.4
Fever	11	83.4	93.0	176.4	16.0	12	103.0	73.4	6.7
Breast Engorgement	8	82.0	0.0	82.0	10.3	4	26.4	55.6	6.9
Dyspnoea	3	7.0	52.0	59.0	19.7	2	32.8	26.2	8.7
Headache	2	0.0	19.0	19.0	9.5	2	7.0	12.0	6.0
Hypertension	4	15.0	1.0	16.0	4.0	3	11.4	4.6	1.2
Leg Swelling	2	14.8	0.0	14.8	7.5	1	2.8	12.0	6.0
Mastitis	2	9.8	3.0	12.8	6.5	2	8.6	4.2	2.1
Heartburn	1	8.0	0.0	8.0	8.0	1	1.2	6.8	6.8
Upper abdominal pain	1	0.0	5.0	5.0	5.0	0	0.0	5.0	5.0
Nausea/Vomiting	2	2.8	0.0	2.8	1.5	1	1.4	1.4	0.7
Dizziness	1	0.6	0.0	0.6	1.0	1	0.4	0.2	0.2
Other	4	42.0	38.0	80.0	20.0	4	52.6	27.4	6.8
Total	161	934.2	1482.0	2416.2	15.0	117	1141.6	1274.6	7.9

LSCS, Lower Segment Caesarean Section

The mean productivity loss adjusted for coping strategies per episode of postpartum ill health in the sample was 7.9 days (SD = 4.4 days). In addition, the mean productivity loss adjusted for coping strategies per episode of pain/infection at LSCS site was 18.8 days and the corresponding figure for an episode of backache was 11.3 days.

### Productivity cost due to postpartum ill health

The productivity cost of the net days lost (without adjusting for the coping strategies) was calculated by taking the net number of days lost due to either partial or total incapacitation multiplied by the mean daily per capita income.

The mean productivity cost per an episode of postpartum ill health in the sample was LKR 5,030.50 (95% CI LKR 3,928.35–6,132.65) equivalent to US$ 34.2(95% CI US$ 26.7–41.6). In addition, the mean productivity cost per an episode of pain/infection at LSCS site was LKR 9,486.16 equivalent to US$ 64.4and the corresponding figure for an episode of backache was LKR 6,469.36 equivalent to US$ 43.9.

The mean productivity cost per an episode of postpartum ill health after adjusting for coping strategies in the sample was LKR 2,653.70 (95% CI LKR 2,070.29–3,235.11) equivalent to US$ 18.0 (95% CI US$ 14.1–21.0). In addition, the mean productivity cost per an episode of pain/infection at LSCS site after adjusting for coping strategies was LKR 6,301.76 equivalent to US$ 42.8and the corresponding figure for an episode of backache was LKR 3,787.76 equivalent to US$ 25.7.

## Discussion

Given the scarcity of literature pertaining to productivity loss and productivity cost in lower middle income countries, the results of the present study provides valuable information in generating the evidence base.

The prevalence of self-reported episodes of ill health among postpartum women in the present study is almost four times higher than the prevalence of postpartum morbidities reported in Sri Lanka in the recent years [10]. Furthermore, the fact that only 16.8% of episodes of ill health necessitated hospitalisation, signifies that hospital based studies to quantify the magnitude of the postpartum ill health would result in gross underestimation of the problem under concern.

Amongst the reported episodes of ill health, the predominant symptoms were lower abdominal pain, backache and pain at episiotomy site and LSCS site. Thus, it highlights the importance of assessing these symptoms which are often overlooked, being attributed as normal phenomena during the postpartum period.

In addition to this high prevalence of postpartum ill health, another noteworthy observation is that, the majority of the episodes of postpartum ill health had a negative impact on household productivity. The aforementioned symptoms were the main contributors of productivity loss, signifying the fact that, though considered as minor ailments, these can cause substantial negative impact on productivity loss. The potential preventability of these minor ailments further emphasises the importance of paying prompt attention to these conditions.

The productivity loss due to postpartum ill health is high with a mean loss of15 days per episode of ill health. Hence, the daily activities of postpartum women are severely restricted including the care for the newborn. Even after adjustment for copying strategies, the productivity loss amounts to almost eight days per episode. The productivity loss within the postpartum period is not only crucial for the mother, but also, critical in relation to the wellbeing of the newborn.

The majority of women who experienced a postpartum illness received assistance, either in the form of intra-household adaptation or through social networks. Assistance through the coping strategies compensated approximately half of the day equivalents lost due to partial and total incapacitation. Albeit the compensation by coping strategies the overall productivity loss is considerable. Not only the majority of day equivalents are uncompensated, but also the substantial proportion of assistance is received from intra-household. These facts signify the increased strain of productivity loss that radiates in the family, beyond the individual postpartum woman.

Furthermore, the estimates of productivity loss could be considered as a probable underestimate of the true magnitude of the problem, given the fact that intra-household compensation would negatively affect the productivity of the household as a single unit, which was not taken into consideration in the present study.

In light of the findings of the present study, the priority areas aimed at reduction of the burden of postpartum productivity loss associated with postpartum ill health are; effective pain management in the postpartum period, infection prevention and control and increased vigilance of health care workers for postpartum “minor ailments” in service provision at institutional and domiciliary care. Hence, this study findings aid in addressing the inequalities in maternal health care service provision, by highlighting the significance of paying more attention on minor ailments, which are otherwise often overlooked.

The total incapacitation consequent to pain and infection at either the LSCS site or episiotomy amounts to 842 day equivalents, representing 34.8% of overall day equivalents loss. This raises the concern that, women experiencing complicated deliveries may require increased postpartum care and surveillance for signs of infection. It is pertinent to ensure that the evidence based practices such as vaginal preparation with antiseptic solutions [19], timing and usage of peri-operative antibiotics [20] are properly in place. In addition, specific focus and increased vigilance on the highlighted symptoms should be paid during postpartum care provision at institutional level by all health care professionals and at domiciliary level by the PHMM, as these are the problems that have a profound effect on women’s life.

The mean productivity cost per an episode of postpartum ill health (without adjusting for the coping strategies) of LKR 5,030.50 accounts for almost one eighth of the mean monthly family income. According to Household Income and Expenditure Survey conducted in 2012/13 in Sri Lanka, the average monthly household expenditure on health and personal care was LKR 2,181.00 [21]. Even the mean productivity cost per an episode of postpartum ill health adjusted for coping strategies (LKR 2,653.70) was higher than this estimate. Another noteworthy observation was that, the mean productivity cost estimates per an episode of easily preventable postpartum ill health conditions such as infection at LSCS site and backache were substantially higher than the reported average monthly household expenditure on health and personal care. Given that during the postpartum period there is a possibility of reduction of the total family income, owing to the fact that women engaged in income generating activities with daily wages are unable to contribute to the income; and increase in the total expenditure of the household, owing to the additional expenses for the newborn and medical expenses for postpartum ill health, the ramifications of the productivity loss would be even more substantial.

Albeit the coping strategies compensated for the productivity cost, as discussed above, the intra-household adaptation could have even increased the productivity cost of the household, if taken into consideration.

### Limitations

Interpretation of evidence from the study should be done considering its limitations. One of the main limitations of the study is the selection bias, i.e., the postpartum women who were hospitalised or having severe limitation of activities for a long duration during the data collection period of the study, were less likely to be included in the study, as the sample recruitment was done at field immunisation clinics. Since the study aimed at assessing the self-reported episodes of postpartum ill health, an inherent association of recall bias is another limitation of the study. This would have resulted in either underestimation or even overestimation of the productivity loss and cost estimates. Nonetheless, measures such as inclusion of women who have delivered within the past 24 weeks, probing for the episodes of ill health in a uniform manner by the interviewers according to a standard protocol and verification of data with documented evidence, whenever appropriate, were taken to reduce the recall bias. However, minor ailments are inherently subjective experiences and would not always be uniformly or completely captured in available treatment records.

In the present study, there were no reported ill health episodes related to mental health issues. The fact that, the majority of health conditions listed in the questionnaire was related to physical health conditions might have hindered participants reporting any associated mental health conditions. Furthermore, some of the well recognised severe postpartum morbidities necessitating prolonged hospitalisation were not captured in this study, as the postpartum mothers who were hospitalized at the time of data collection were not included in the study.

The study was conducted in 2011, hence while extrapolating the study findings to the current day settings, it is noteworthy to be cognizant of the differences of health care resources, use and costs.

Owing to the heterogeneity of methodologies used in productivity cost estimation in studies, it is important to appreciate the cost estimation techniques while comparing results across different studies. When interpreting the productivity cost estimates, it is crucial to appreciate the contextual differences in the expected contribution of the postpartum mothers to the household productivity. The productivity cost estimates of the present study were computed in the context where a postpartum mother is expected to be mainly engaged in providing care to the newborn and also a female employee is eligible for a 12 weeks (84 days) paid maternity leave, according to the Maternity Benefits Ordinance in Sri Lanka. Hence, the cost estimates of the study should be extrapolated to different settings with caution.

## Conclusions

The prevalence of postpartum morbidity was high among postpartum women in Anuradhapura district, with easily preventable conditions such as lower abdominal pain, backache and pain and infection at LSCS site and episiotomy site being the predominant causes of ill health. The mean productivity loss due an episode of to postpartum ill health was high, even after adjusting for coping strategies. The mean productivity cost per an episode of postpartum ill health in the sample was US$ 37.7 (95% CI US$ 29.4–45.9) and the mean productivity cost per an episode of postpartum ill health after adjusting for coping strategies in the sample was US$ 19.9 (95% CI US$ 15.5–24.2). The mean productivity costs per an episode of infection at LSCS site and an episode of backache were even higher. Thus, it is recommended to pay more attention in the aspects of provision of effective pain management in the postpartum period, evidence-based infection prevention and control and increased vigilance of health care workers for postpartum “minor ailments” in service provision at institutional and domiciliary care, to reduce the burden of postpartum productivity loss associated with postpartum ill health.
